# Seroprevalence of Rift Valley fever in cattle along the Akagera–Nyabarongo rivers, Rwanda

**DOI:** 10.4102/jsava.v88i0.1379

**Published:** 2017-01-20

**Authors:** Thérèse Umuhoza, Dirk Berkvens, Isidore Gafarasi, Joseph Rukelibuga, Borden Mushonga, Savino Biryomumaisho

**Affiliations:** 1 Department of Biomedical Sciences, Institute of Tropical Medicine, Belgium; 2 Veterinary Services Unit, Rwanda Agriculture Board, Rwanda; 3 Centers for Disease Control and Prevention, Kigali, Rwanda; 4 Department of Biomedical Sciences, University of Namibia, Namibia

## Abstract

Rift Valley fever (RVF) virus is caused by a zoonotic arbovirus that is endemic to eastern and southern Africa. It has also been reported in West and North Africa, Madagascar and the Arabian Peninsula. The virus is transmitted by mosquitoes, but people can also become infected while handling blood or other body fluids of animals and humans with RVF. In 2007, there was a large outbreak of RVF in Kenya, Tanzania, Sudan and Somalia. Outbreaks were also reported in South Africa in 2008–2011. The epidemiology of RVF and factors for disease occurrence in Rwanda are neither clear nor documented. Therefore, we conducted a cross-sectional study from December 2012 to March 2013 to generate baseline information on RVF in cattle. Purposive sampling of cattle (*n* = 595) was done in six districts, and serum samples were screened with competitive enzyme-linked immunosorbent assay (ELISA). We performed a statistical analysis on the generated data, and risk factors associated with RVF seroprevalence were determined by a simple logistic regression. Overall, RVF seroprevalence was 16.8% (95% confidence interval [CI] [13.8% – 20.0%]). The highest seroprevalence was recorded in Kirehe district (36.9%) followed by Ngoma (22.3%), and the least was recorded in Nyagatare (7.9%). RVF was more likely to occur in adult cattle (19.9% [odds ratio {OR} = 1.88, 95% CI {0.98–3.61}]) compared to young cattle (10.5% [OR = 0.47, 95% CI {0.26–0.83}]). Pure exotic or cross-breeds were significantly exposed to RVF virus (seroprevalence 22.9% [OR = 4.26, 95% CI {1.82–9.99}]) in comparison to 14.1% (OR = 0.55, 95% CI [0.35–0.86]) in local breeds. Sex differences were not statistically significant. These findings indicated that cattle have been exposed to RVF virus in six districts in Rwanda with a significant risk in adult, exotic or cross-breeds in Kirehe district.

## Introduction

Rift Valley fever virus (RVFV) was initially isolated at Kabete, Kenya, in 1930 (Daubney, Hudson & Garnham 1931). Many outbreaks have since been described and the disease has been associated with heavy rainfall and prolonged rainy seasons (Anyamba et al. 2009; Davies 2010). Rift Valley fever (RVF) is caused by an arbovirus of the family Bunyaviridae. The virus is transmitted by infected mosquitos (Anyamba et al. 2009). It is widely spread in Africa from Egypt to South Africa, and the disease has been reported in Madagascar, Saudi Arabia and Yemen (Balenghien et al. 2013; Boshra et al. 2015; Swanepoel & Coetzer 2004). RVF continues to threaten public health throughout Africa, with risks of it spreading globally (Bird et al. 2009). In ruminant species, specifically sheep and cattle, the disease is recorded to cause up to 100% mortalities in neonatal animals and extensive abortion storms in infected pregnant animals (Bird et al. 2009; Coetzer 1977, 1982). In humans, the infection is described as a febrile disease. However, complications such as retinitis, blindness and encephalitis have been reported in 1% – 2% of affected individuals (Madani et al. 2003).

In eastern and southern Africa, RVF epidemics occur at irregular intervals of 5–15 years (Anyamba et al. 2009; Davies & Martin 2003). After flooding, vector-competent mosquitoes such as *Aedes* and *Culex* spp. lay their eggs in floodwater, which results in an increased population of RVF vectors and the subsequent transmission to vertebrate hosts (Anyamba, Linthicum & Tucker 2001). However, the outbreaks can be forecast by monitoring of climatic parameters and early detection of viral circulation through active surveillance (Davies & Martin 2003).

The prediction map of eastern and southern Africa indicates an increased risk of the spread of RVF because of climate change, which can facilitate increased numbers of vectors (Anyamba et al. 2009). However, the epidemiology of RVF and the factors prompting disease occurrence in Rwanda are neither clear nor documented. It is vital to appreciate that RVF is an important transboundary disease whose epidemiology and zoonotic potential must be understood in the context of designing effective control measures. This should assist in creating an environment conducive to local and international livestock trade, improved food quality, food security and public health. The present study aimed at generating baseline information on seroepidemiology of RVF in cattle reared along the Akagera–Nyaborongo rivers in the Nile basin of Rwanda.

## Materials and methods

### Study area

Geographically, the dense hydrological network of Rwanda consists of 278 536 ha covered by lakes, rivers and marshlands with a large portion (83%) located in the Nile basin. The Akagera and Nyabarongo rivers drain 90% of the country’s water bodies. Rwanda’s temperature changes with the landscape with an overall average of 20 °C. The temperature of the eastern plateaus of Rwanda is on average 20 °C – 21 °C; in the southern-west valley of Rusizi (Impara and Imbo), temperatures average between 23 °C and 24 °C while temperatures of 17.5 °C – 19.0 °C on average are recorded for Buberuka highland and the central plateau. Temperatures of less than 17 °C are recorded for the Congo ridges, which stretch up to the high volcanoes (Safari 2012). The country’s annual rainfall varies, and the differences range from more than 1200 mm in the elevated zone of the north-west to less than 900 mm in the eastern zone (REMA 2011; Safari 2012). Livestock production has been an integral part of the farming system in Rwanda, predominantly in the Nile basin. Data from the Integrated Household Living Condition Survey of 2008 reported that 70% of the households own livestock.

### Sources of data

The data in this study were provided by the National Veterinary and Laboratory Services Unit in Rwanda. Epidemio-surveillance was conducted in six districts, namely, Bugesera, Nyagatare, Ngoma, Kirehe, Gakenke and Kamonyi located along the Akagera–Nyabarongo rivers in the Nile basin from December 2012 to March 2013. The districts were purposively sampled, following reports of abortion storms in cattle. Information compiled in the data set included laboratory results of the screened population. A total of 595 bovine serum samples were tested for antibodies against RVFV with a c-ELISA (enzyme-linked immunosorbent assay) kit (ID screen^®^ Rift Valley fever competition multispecies ELISA, ID-VET, Monpellier, France). Breed types were recorded as local, pure exotic or cross-breeds. Two categories of age, young (< 12 months) and adult (≥ 12 months), were reported. Sex and test results were provided in the same data set. The vector layers of Rwanda (administration boundaries, rivers, lakes and basin) were collected from the library of Rwanda at the Global Agriculture and Food Security Program (GAFSP) website.

### Data analysis

The data set received in Microsoft Office Excel 2010 was cleaned and verified for errors. Data analysis was performed with STATA^®^13 (STATA Corporation, College Station, TX, USA). RVF seroprevalence (P) was measured as the proportion of the number of animals presenting antibodies against RVFV (exposed group) in the tested population. The estimate of proportion was computed with an exact 95% confidence interval (CI) based on the probabilities delivered from a binomial distribution. Simple logistic regression was appropriately used to test the relationship of RVF seroprevalence (outcome) and associated risk factors (exposure or predictors variable). Univariate logistic regression was performed to examine the relationship between each individual predictor (locality, breed, age and sex) and RVF seroprevalence. The variables with *p* < 0.1 were considered to be potentially covariate and they were included in multivariable analysis. Variables were considered statistically significant when *p* < 0.05. The forward selection approach was used to build the final model. Potential confounders were evaluated during the process. A variable was considered as a confounder when the coefficients of primary variable (e.g. locality) changed substantially (i.e. > 25%) while adding a confounding variable in the model. Two-way interactions were checked through the process by adding a cross product term (##) in the model. The final model was confirmed by the mean of Akaike information criterion (AIC), and the model with the lowest AIC was considered the best model. The spatial distribution of RVF seroprevalence was visualised in the Nile basin by joining the layer of Rwanda’s boundaries with the estimate of RVF seroprevalence in the QGIS (Quantum GIS.2.0.1- Lisboa).

## Results

Overall RVF seroprevalence was estimated to be 16.8% (*n* = 595) at 95% CI (13.8% – 20.0%). A seroprevalence of 36.9% was recorded in Kirehe district with 95% CI (25.9–49.0) followed by Ngoma (22.3%, 95% CI [14.3% – 32.0%]), Bugesera (17.8%, 95% CI [10.9–26.6]) and Kamonyi (14.4, 95% CI [8.1% – 23.0%]). In the districts of Gakenke and Nyagatare, RVF seroprevalence was 9.8% (95% CI [4.6% – 17.9%]) and 7.9% (95% CI [4.0% – 13.7%]), respectively (Table 1).

**TABLE 1:** Potential risk factors associated with Rift Valley fever seroprevalence based on univariate logistic regression.

Variable	Level	*n*	% Positive	95% CI*	*p*
Locality	Kirehe	73	36.9	25.9–49.0	< 0.001
	Bugesera	101	17.8	10.9–26.6	
	Kamonyi	97	14.4	8.1–23.0	
	Gakenke	91	9.8	4.6–17.9	
	Ngoma	94	22.3	14.3–32.0	
	Nyagatare	139	7.9	4.0–13.7	
Age	Young	152	10.5	6.1–16.5	0.006
	Adult	402	19.9	16.1–24.1	
Breed	Local	326	14.1	10.5–18.3	0.009
	Pure exotic or cross	209	22.9	17.4–29.2	
Sex	Male	62	11.2	4.6–21.8	0.166
	Female	500	18.0	14.7–21.6	

*Source*: Department Biomedical Sciences, Institute of Tropical Medicine, Antwerp, BelgiumYoung, animal’s age < 12 months; adult, animal’s age > 12 months.

*n*, sample size; CI, confidence interval.

* reference level.

The risk of RVF occurrence was significantly higher in Kirehe district compared to all other districts (Table 2). RVF seroprevalence was significantly different between the breeds, with *p* = 0.001 (22.9%; [OR = 4.26]) in the exotic or cross-breeds versus (14.1%; [OR = 0.23]) the local breed. In adult cattle, RVF seroprevalence was estimated at 19.9% (OR = 1.88), while in young animals it was 10.5% (OR = 0.53). The difference in RVF seroprevalence noted in the bovines’ age was statistically significant with *p* = 0.05, whereas the sex difference was not statistically significant with a seroprevalence of 18% and 11.2% in female and male cattle, respectively.

**TABLE 2:** Rift Valley fever seroprevalence after adjusting with locality, age, breed and sex in multivariate analysis.

Variable	Level	*n*	% Positive	OR	95% CI*
Locality	Kirehe	73	36.9	1*	-
	Bugesera	101	17.8	0.005	0.00–0.04
	Kamonyi	97	14.4	0.013	0.00–0.11
	Gakenke	91	9.8	0.005	0.00–0.04
	Ngoma	94	22.3	0.034	0.04–0.28
	Nyagatare	139	7.9	0.004	0.05–0.03
Breed	Local	326	14.1	1*	-
	Pure exotic or cross	209	22.9	4.26	1.82–9.99
Age	Young	152	10.5	1*	-
	Adult	402	19.9	1.88	0.98–3.61

*Source:* Department Biomedical Sciences, Institute of Tropical Medicine, Antwerp, BelgiumYoung, animal’s age < 12 months; adult, animal’s age > 12 months.

*n*, sample size; OR, odds ratio; CI, confidence Interval.

* reference level.

## Discussion

**FIGURE 1:**
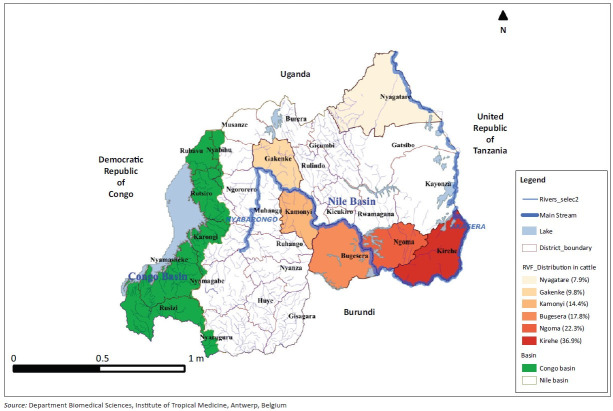
Distribution of Rift Valley fever seroprevalence along the Akagera–Nyabarongo rivers in the Nile basin.

The Nile basin of Rwanda has suitable ecological conditions for RVFV circulation including support for vector survival. Typically, the Nile basin is drained by two large rivers known as the Akagera–Nyabarongo network and it has widespread swamps and numerous dambos that harbour sites for breeding mosquitoes. In this study, the overall seroprevalence of RVF in cattle was estimated to be 16.8% (*n* = 595) along the Akagera–Nyabarongo rivers in the Nile basin. This figure is close to the findings of 13.1% (*n* = 1396) seroprevalence reported in cattle in Ijara district, Kenya (Owange et al. 2014). It was also high compared to the seroprevalence of 11.3% (*n* = 970) reported in cattle in the Kilombero river valley in Tanzania (Sumaye et al. 2013), but lower than findings of 38.7% in cattle sampled from other areas in Tanzania (Chengula et al. 2014). This variation could be because of the differences in sampling techniques, the diagnostic test used or the agro-ecological conditions. The distribution of RVF seroprevalence varied among districts (Figure 1), with high seroprevalence noted in Kirehe (36.9%) and Ngoma (22.3%) districts, which are located in the eastern plateau bordering northern Tanzania.

The differences may be attributed to the variation of competent mosquito vectors because of the extensive swamps. In addition, the river marks the northern border of Tanzania, which is a national park. Contact with wildlife has been identified as an important RVF transmission pathway risk factor (Owange et al. 2014).

The highest seroprevalence (36.9%) recorded in Kirehe district can be attributed to the physical characteristics of the district: temperate climate and low altitude, swamps, paddy rice fields and other crops, and dense vegetation with a large variety of *Acacia* trees. These factors make the area suitable breeding sites for crepuscular and nocturnal mosquitoes such as *Aedes, Culex* and *Anopheles* genera. Such an ecosystem of temperate environment, thick rainy forest and low altitude has been associated with the occurrence and transmission of the RVFV in Madagascar (Chevalier et al. 2008). It could also be as a result of some activities in the area including livestock trade, as it has been established that animal movements are linked to the introduction of RVFV into new areas. This was evidenced in Egypt during the 1977 outbreak (Shope, Peters & Davies 1982). In 2011, a study was conducted in Kigoma rural district near Lake Tanganyika at the border of Tanzania with Burundi, where RVF seroprevalence was estimated at 12% and the report suggested that animal movements in the region may have played an important role (Kifaro et al. 2014). This has also been corroborated by Jeanmaire et al. (2011) in Madagascar, who observed an anti-RVFV IgG seroprevalence of 28.1% in bovines procured to replenish herds. Older cattle were found to be at a higher risk of having circulating anti-RVF antibodies in this study. This was attributed to increased duration of life and the concomitant increased risk of being infected with RVFV (OR = 1.88, 95% CI [0.98–3.61]). Increased risk in older animals suggests that persistent exposure over time is an important factor in the high risk areas. These findings are in agreement with other study reports conducted in Mauritania (Fontenille et al. 1998), Madagascar (Jeanmaire et al. 2011) and Tanzania (Sumaye et al. 2013). The study noted that the animal’s age was naturally the indicator of RVF endemicity in the area, where young animals are less exposed than older animals. The high risk noted in pure exotic breeds (OR = 4.26, 95% CI [1.82–9.99]) could be explained by the high susceptibility of exotic breeds to RVFV compared to local breeds. With regard to animal movements, cattle importations increased remarkably in the past 5 years in Rwanda. According to FAOSTAT, the number of imported cattle was 2943 in 2008, 4035 in 2009, 3936 in 2010, 4763 in 2011 and 6692 in 2012. In addition, Rwanda through its *One Cow per Poor Family* programme imports considerable numbers of cattle for distribution to the poor families. It has been postulated that local breeds are naturally less susceptible to clinical diseases (Davies & Martin 2003), but they are also known to be less productive in the development programme in the region.

## Conclusion

Our findings indicate that cattle in districts along the Akagera–Nyabornongo rivers were exposed to RVFV, with a high risk in Kirehe district. In addition, the exotic breeds are more likely to get the infection (OR = 4.26) and the risk increases in the older animals (OR = 1.88). Taking into account that the populations in the area (70%) are household farmers who live in close contact with the animals, and that they live in an ecosystem suitable for the circulation of RVF vectors, there is a high risk of transmission of RVF to humans. Therefore, a One Health approach is necessary for the control of the disease.
